# Physiological and Proteomic Responses of Sugarcane to Water Deficit Stress: Insights from a Self-Fertilized Clone

**DOI:** 10.3390/ijms262311571

**Published:** 2025-11-28

**Authors:** João de Andrade Dutra Filho, Adauto Gomes Barbosa Neto, Cinthya Mirella Pacheco Ladislau, Marcelle Almeida da Silva, Geisenilma Maria Gonçalves da Rocha, Rômulo Gil de Luna, Anielson dos Santos Souza, Lauter Silva Souto, Ancélio Ricardo de Oliveira Gondim, Andréa Chaves Fiuza Porto, Fabiana Aparecida Cavalcante Silva, Josimar Mendes de Vasconcelos, Guilherme Rocha Moreira, Diogo Gonçalves Neder, Francisco Cássio Gomes Alvino, Leonardo de Sousa Alves, Lucas Carvalho de Freitas, Djalma Euzébio Simões Neto, Marcelo Menossi, Tercilio Calsa Junior

**Affiliations:** 1 Biological Science Nucleus, Vitoria Academic Center, Federal University of Pernambuco, Recife 55608-680, Brazil; 2 Institute of Biological Sciences, University of Pernambuco, Recife 50100-130, Brazil; adauto.barbosa@upe.br; 3 Postgraduate Program Rede Nordeste de Biotecnologia, Federal Rural University of Pernambuco, Recife 52171-900, Brazil; cinthya.m.pacheco@gmail.com; 4 Biological Sciences Course, Agricultural Sciences Campus, Federal University of Vale do São Francisco, Petrolina 56304-917, Brazil; marcelle.almeida@univasf.edu.br; 5 Postgraduate Program Genetic, Federal University of Pernambuco, Recife 50670-901, Brazil; geisenilmarocha@gmail.com; 6 Agri-Food Science and Technology Center, Federal University of Campina Grande, Pombal 58840-000, Brazil; romulo.gil.luna@gmail.com (R.G.d.L.); anielsonsantos@gmail.com (A.d.S.S.); lautersouto@yahoo.com.br (L.S.S.); anceliogondim@gmail.com (A.R.d.O.G.); 7 Dom Agostinho Ikas Agricultural College, Federal Rural University of Pernambuco, São Lourenço da Mata 54735-000, Brazil; achavesfiuza@yahoo.com.br; 8 Phytosanitary Diagnosis and Fidelity Genetic Laboratory, Northeast Strategic Technologies Center, Avenida Professor Luís Freire, Cidade Universitária, Recife 50740-545, Brazil; fabiana.acs@gmail.com; 9 Department of Statistics and Informatics, Federal Rural University of Pernambuco, Recife 52171-900, Brazil; josimar.vasconcelos@ufrpe.br (J.M.d.V.); guirocham@gmail.com (G.R.M.); 10 Department of Agroecology and Agriculture Center for Agricultural and Environmental Sciences, University of Paraíba, Sítio Imbaúba, sn, Zona Rural, Lagoa Seca 58429-500, Brazil; dgneder@hotmail.com; 11 Department of Agricultural Engineering, Federal University of Viçosa, Viçosa 36570-900, Brazil; cassioalvin2@gmail.com; 12 Department of Plant Sciences Federal, Rural University of the Semiarid, Mossoró 59625-900, Brazil; leo_agro22@hotmail.com; 13 Postgraduate Program Genetic, Universidade Federal de Pernambuco, Recife 50670-901, Brazil; lufcas@gmail.com; 14 Sugarcane Experimental Station of Carpina, Sugarcane Breeding Program, Federal Rural University of Pernambuco, Carpina 52171-900, Brazil; djalmasimoesneto@gmail.com; 15 Center for Plant Molecular Breeding, State University of Campinas, Campinas 13083-862, Brazil; menossi@lgf.ib.unicamp.br; 16 Laboratory of Plant Genomics and Proteomics, Department of Genetics, Biosciences Center, Federal University of Pernambuco, Recife 50670-901, Brazil; tercilio.calsaj@ufpe.br

**Keywords:** climate changes, drought, environmental stress, plant breeding, plant tolerance

## Abstract

Abiotic stresses, particularly water deficit, are major constraints to global agricultural productivity. This study aimed to evaluate physiological and proteomic responses in two sugarcane genotypes, a cross-commercial cultivar and a self-fertilization clone, subjected to water deficit stress in the field. The experiment was conducted under rain-fed conditions. Organic solutes, photosynthetic pigments, gas exchange, and the quantum efficiency of photosystem II were evaluated. Total protein was extracted using the phenol method, and the peptides were analyzed using mass spectrometry. Elevated proline levels in clone RB061047 suggest a potentially enhanced adaptive response to water-deficit stress. There were no marked differences in the photosynthetic pigments between clone RB061047 and the commercial cultivar, RB867515. Self-fertilization did not negatively affect the physiological performance of RB061047 under water-deficit conditions because the higher photosynthetic rate and the consequent more efficient use of water suggest a marked gain in biomass and productivity. The ATP synthase alpha subunit YABB2 protein, fructose-bisphosphate aldolase, and nucleoside diphosphate kinase 1 emerged as potential candidates for the development of functional molecular markers for the selection and development of new sugarcane cultivars that are more tolerant to water-deficit stress.

## 1. Introduction

Environmental or abiotic stresses are one of the main factors limiting agricultural productivity worldwide [1]. Stress refers to any environmental factor that disrupts normal physiological functions and reduces plant performance [2]. The most common abiotic stressors include salinity, water deficit, nutrient deficiency or excess, and high and low temperatures [3]. Water deficit occurs when cellular water content falls below the threshold required to maintain essential physiological processes [4]. According to Ferreira et al. [5], water deficit is one of the most important factors limiting sugarcane production, especially in areas with a prolonged period of drought.

The annual water consumption of sugarcane is 1500–2500 mm [6]. However, the irregular distribution and reduced rainfall in the state of Pernambuco during vegetative growth of the crop cause substantial losses, with a sharp decline in productivity and mortality of the ratoons, forcing early renewal of the sugarcane field [7]. Irrigation is often essential for mitigating the impact of water deficits on sugarcane production systems [8].

In the sugarcane region of Pernambuco State, water availability is limited to the central, northern Atlantic, and northern coastal micro-regions. The use and costs of irrigation equipment are very expensive. The most viable alternative is to develop improved cultivars with high productivity and tolerance to water deficit stress [9].

Tolerance to water deficit stress can be defined as the ability of plants to acclimate to unfavorable water-deprived conditions [10]. The acclimatization process in plants subjected to water-deficit stress begins with stress perception, followed by a complex sequence of molecular events, culminating in various physiological, metabolic, and developmental responses [11].

Under water-deficit stress conditions, morphophysiological responses generally include stomatal closure [12], increased synthesis of osmoprotectants [13,14], decreased efficiency of photosystem II quantum yield [15], and decreased stomatal conductance and photosynthetic rate [16].

Water deficit is one of the environmental stressors responsible for changes in leaf pigments [17]. Therefore, the assessment of these physiological parameters and the estimation of these pigments should be used to select genotypes tolerant to drought [18,19]. Furthermore, the identification of specific proteins involved in stress perception can elucidate the tolerance mechanisms and the development of new cultivars with high productivity under unfavorable cultivation conditions.

To develop new sugarcane cultivars tolerant of environmental stresses, biparental crosses have been used, in which two elite parents with a gene set of interest are intercrossed. This type of crossing is most commonly used and aims to exploit heterosis [20].

As a predominantly allogamous species, the effects of inbreeding, especially in its most extreme form, self-fertilization, should be avoided in sugarcane. This practice usually promotes inbreeding depression and the subsequent loss of vigor and yield productivity, rendering individuals susceptible to environmental variation and unviable for commercial cultivation [21]. Inbreeding depression is associated with deleterious and lethal alleles in the homozygous genotypes. The expression of several recessive alleles remains hidden under heterozygous conditions in natural populations. As homozygosity increases in inbred populations, there is a higher probability for recessive traits to manifest, resulting in the loss of vigor and productivity [22].

Depression may be substantial in respect of quantitative traits, such as lower germination, slower growth, lower weight and number of fruits, and lower grain yield [23]. Additionally, changes in physiological processes, such as decreased photosynthetic pigment biosynthesis and photosynthesis rates, have been observed. This occurs mainly because all these processes are enzymatically controlled, and an increase in the number of loci in recessive homozygosity, coding for less functional enzymes or expressed at low levels, may lead to disrupted metabolic pathways and impair plant development [24].

However, it has been observed that the self-fertilization of sugarcane does not always lead to a decrease in vigor in its descendants. A notable example is the Argentinian cultivar, NA 56-79, derived from the self-fertilization of ‘Co 419’. It was extensively cultivated in Brazil during the 1970s–1980s and achieved the highest sugar yield per hectare among several other cultivars grown in the state of São Paulo. Numerous other cases have been reported in which self-fertilization did not result in inbreeding depression, producing cultivars with good performance [25].

Given the above context, the objective of this study was to evaluate the effects of water deficit stress on the biochemical and physiological processes of the RB061047 self-fertilizing sugarcane clone and RB867515, a commercial hybrid, under field conditions. The complementary identification of differentially accumulated proteins after stress perception was added to elucidate the potential elements involved in tolerance mechanisms.

## 2. Results

### 2.1. Soil Analysis

The soil had a predominantly sandy texture, which resulted in low water retention. The available water was considered low for all samples (<0.05 cm^3^/cm^3^), an expected result for soils with a high proportion of sand, which have macropores that quickly drain water (Table 1). 

### 2.2. Organic Solutes

Regarding the biochemical analysis, genotypes RB867515 and RB061047, subjected to water deficit stress, exhibited distinct behaviors regarding the accumulation of carbohydrates and proline (Table 2).

### 2.3. Photosynthetic Pigments

Regarding the chlorophyll and carotenoid content (Table 2), there were no marked differences among the treatments, indicating that they essentially exhibited the same behavior in the accumulation of photosynthetic pigments.

### 2.4. Gas Exchange

Statistical differences were observed among the treatments in terms of photosynthesis and water-use efficiency (Table 2).

### 2.5. Quantum Efficiency of Photosystem II

In the analysis of the variables observed in the chlorophyll a fluorescence measurements, statistical differences were observed between the initial and maximum fluorescence (Table 2).

### 2.6. Proteomic Analysis

**Figure 1:**
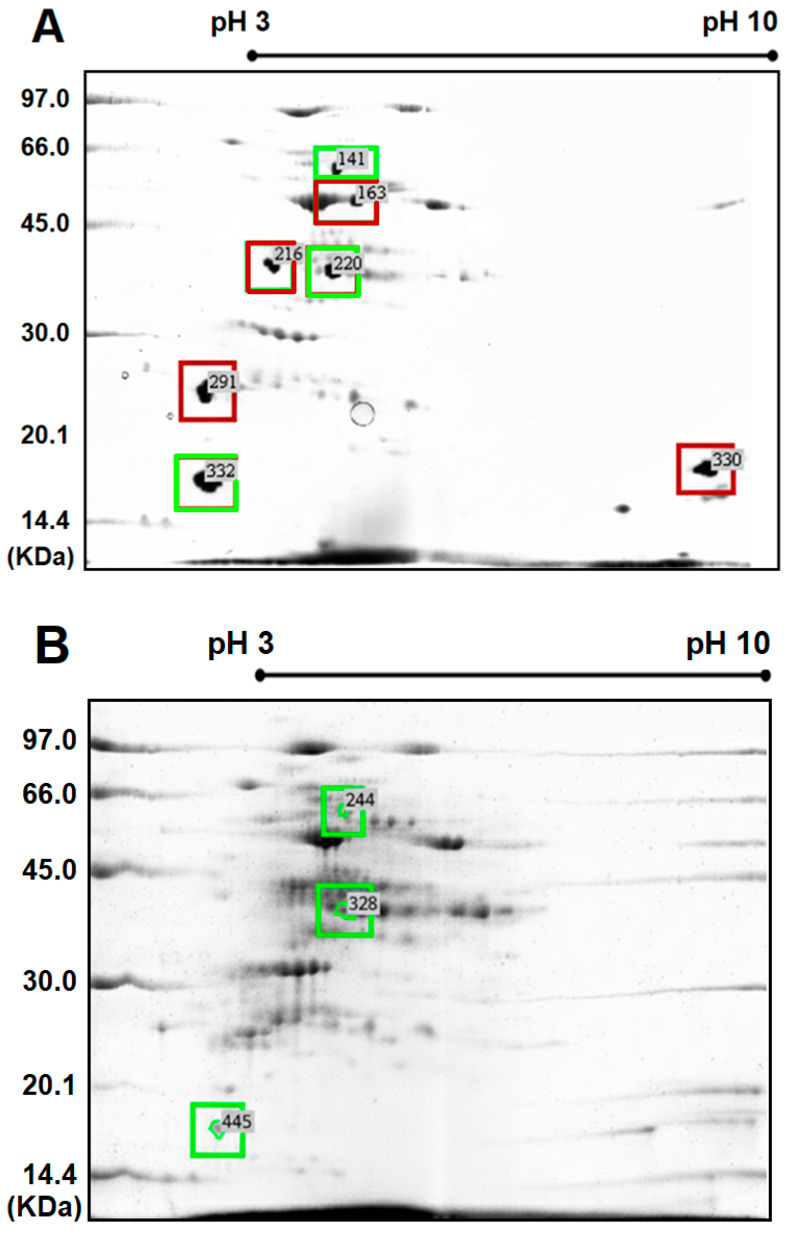
Representative images of two-dimensional polyacrylamide gel electrophoresis (2D-PAGE) gels (12.5% polyacrylamide) for total proteins extracted from the +1 leaf of sugarcane cultivated under field conditions subjected to water deficit stress. (**A**) 2D proteome of the commercial cultivar, RB867515 (a hybrid tolerant to water deficit stress). (**B**) 2D proteome of the clone, RB061047, derived from the self-fertilization of RB061047. Spots highlighted in red refer to proteins expressed exclusively in the commercial cultivar, RB867515. Spots highlighted in green refer to proteins expressed in both genotypes.

Extraction proved to be efficient, as a satisfactory amount of protein was successfully extracted from both treatments for subsequent two-dimensional (2D) analysis. The mean values of protein concentration from the three biological replicates were 0.400–0.814 μg. μL^−1^. The extracted proteins were found to be intact and of excellent quality, and 2D gels were created from three biological replicates per treatment (Figure 1).

In this bi-dimensional proteomics approach presented on 2D gels, similar proteomic profiles were observed between the evaluated genotypes. A total of 689 spots (proteins) were assigned: 281 from the clone derived from self-fertilization and 408 from the commercial cultivar, RB867515. Proteomic image comparison resulted in a total of seven spots selected as differential, based on a percentage volume variation ratio ≥ 1.5 and analysis of variance *p*-value ≤ 0.05. Of these, three were detected in the proteome of both genotypes under water-deficit conditions, whereas four proteins were exclusively found in the proteome of the commercial cultivar, RB867515.

Seven spots selected for digestion and protein identification using mass spectrometry were successfully identified. Their amino acid sequences were putatively obtained from *m*/*z* spectra using the positive reflected method (PMF), compared with the database, and showed a high correlation with plant polypeptides (Table 3).

## 3. Discussion

The soil analysis indicated a predominantly sandy texture, which resulted in low water retention and nutrient leaching. For plants, low water retention in the soil indicates that they face a much higher risk of water stress and require careful management [26]. This statement can be corroborated by the rainfall index of only 511.6 mm during the period in which the experiment was conducted. Sugarcane requires 1500–2500 mm of well-distributed rainfall for its full development, thus confirming the occurrence of a water deficit [6].

Cultivar RB867515 accumulated a higher amount of carbohydrates than clone RB061047. The RB867515 cultivar exhibits rapid growth as one of its characteristics when grown under conditions of water-deficit stress [27]. The accumulation of carbohydrates in this cultivar justifies the need for energy for its rapid development. Similar results were reported by Sales et al. [28]. These authors observed an increase in carbohydrate levels in sugarcane subjected to water-deficit stress.

According to Bray [29], carbohydrates (glucose and fructose) are among the most important osmoregulators, and the enzymes involved in their synthesis allow for osmotic adjustment or net accumulation, resulting in a decrease in osmotic potential. This helps maintain water flow in favor of a water potential gradient, protecting against leaf turgor [30]. Supporting this understanding, Pimentel [31] reported that osmotic adjustment due to the work of these osmoregulators promoted the necessary turgor for plant growth under the evaluated stress conditions.

RB061047, derived from self-fertilization, exhibited a substantially higher proline accumulation than RB867515. Proline is an osmoprotector that can be used as a biochemical marker for cultivars subjected to water deficit stress [32]. Under the influence of water-deficit stress, protein synthesis is inhibited and degradation is rapidly accelerated, leading to a considerable increase in amino acids and free amines. This causes severe disturbances in protein metabolism. A change in amino acid proportions and a marked increase in proline concentration result in an adaptive response of plants to water deficit stress [33,34].

Bray [29] and Mittler [33] also reported that the role of proline in plants was to serve as a reserve of organic nitrogen, which can be used during rehydration and the synthesis of specific proteins for acclimatization under stressful conditions.

The increase in proline levels is linked to an increase in the biosynthesis of this amino acid and would be a protective mechanism against water deficiency [35]. This amino acid promotes the reduction in tissue water potential, maintains tissue integrity, and retains water [36]. This was observed in the analysis of gas exchange, where the RB061047 clone showed higher water-use efficiency and greater photosynthetic activity, as it did not degrade chlorophylls and carotenoids.

Chlorophylls a and b, as carotenoids, are the most abundant biological pigments on the planet, and because of their chemically unstable structures, they can be easily degraded, especially if plants are under environmental stress [37]. This degradation results in decomposition products that modify the perception and quality of the plants. One consequence of endogamous depression, in addition to the loss of vigor and decline in productivity, is the emergence of albino plants, which have a drastic reduction in photosynthetic pigments and, consequently, a marked decrease in the photosynthetic rate [38,39].

However, the results of the current study clearly demonstrate that self-fertilization does not cause undesirable alterations in the physiological processes of the RB061047 clone. The higher photosynthetic rate and the concomitant more efficient water-use suggest a marked increase in biomass and productivity [8,40,41]. Greater tolerance to water-deficit stress was evident, considering that the plant used the limited water available in the soil more efficiently, as shown in the soil physical analysis.

Regarding the quantum efficiency of photosystem II, both the commercial cultivar, RB867515, and the clone resulting from self-fertilization, RB061047, maintained high values, with no statistical difference. This is indicative of the efficiency of radiation use and a relatively rapid response to water-deficit stress, which is an important trait in selecting new drought-tolerant sugarcane genotypes for improvement [18]. The results during the stress period indicate that the genotypes exhibited high recovery from temporary photoinhibition damage in photosystem II. From a biochemical and physiological standpoint, the clone resulting from self-fertilization proved superior to the commercial cultivar in terms of important traits for selecting genetic materials with enhanced tolerance to water deficit stress.

The proteomic analysis provided complementary and relevant information for understanding these biochemical and physiological responses, potentially contributing even more to breeding programs for the development of improved cultivars with tolerance to water-deficit stress. In the proteomic analysis, the identification of a small number of proteins, specifically seven spots, was expected. The non-significant ANOVA, when applied in proteomic analysis, should not be interpreted as being influenced by experimental error, but rather as a strong indication that the expression levels of many common and unique proteins in the considered treatments were not significant. This is because, in the case of self-fertilization, the generated genetic variability is lower than that resulting from biparental and polycross crossings. In other words, differences in the genetic constitution of the clone resulting from self-fertilization were not due to chromosomal shuffling (metaphase I and anaphase I of meiosis I) of the two distinct parents [42]. Instead, they arose from chromosomal rearrangements within the same individual; in this case, cultivar RB867515. Through self-fertilization, RB867515 simultaneously acts as both the male and female parent, giving rise to a clone and increasing the probability of fixing new polyploid lineages [43]. Therefore, the difference in protein expression levels and the number of exclusive proteins should be substantially reduced because of the genetic similarities between the genotypes.

In this way, self-fertilization, in the first generation, promotes an increase in the number of loci in homozygosity, consequently generating lower genetic and allelic variability [44]. Differentially expressed proteins persist under stress conditions and should be studied with caution when selecting and developing genotypes tolerant to abiotic stresses. In the present study, the results are relevant and interesting for breeding, as proteins playing an important role in the mechanism of tolerance to water-deficit stress were identified.

The alpha subunit of ATP synthase and fructose-bisphosphate aldolase were identified by Pacheco et al. [45]. ATP synthase alpha subunit (ATPA) is encoded by the *atpA* gene located in the plastome and is present in chloroplasts, specifically in the thylakoid membrane. It produces adenosine triphosphate (ATP) from ADP in the presence of a proton gradient across the membrane [46]. These authors also emphasized the importance of ATPA in removing toxic ions from the cytoplasm and organelles of plants under stress conditions.

The protons produced by water oxidation, with those transferred by plastoquinone, generate an electrochemical potential within the thylakoids used in ATP synthesis [47]. Thus, photosystem II depends on water to generate the chemical energy required for CO_2_ fixation. With lower water availability, less ATP and NADPH are formed, and less CO_2_ is fixed [48,49]. Therefore, a smaller amount of water results in lower efficiency of photosystem II. Consequently, the higher expression of ATP synthase produced by the clone resulting from self-fertilization indicates that it utilizes the limited water availability in its environment much more efficiently, as demonstrated by physiological data, producing more energy for metabolism and development.

The presence of ATP synthase implies energy supply to plants under stress conditions. Janpromma et al. [50] analyzed the proteome of commercial sugarcane cultivars under water-deficit stress and identified the presence of ATP synthase, which plays an important role in maintaining chloroplast function. They further mentioned that ATP synthase responded consistently to water-deficit stress conditions. Therefore, the higher expression of this protein in the self-fertilized clone is also indicative of efficient growth and development of available energy under water and salt stress. In light of the aforementioned facts, ATPA proves to be a highly promising molecular marker in the development of functional molecular markers.

Fructose-bisphosphate aldolase (FBA) is a protein present in chloroplasts, cell walls, and cytosol. This enzyme converts fructose 1,6-bisphosphate into glyceraldehyde 3-phosphate and dihydroxyacetone [51]. FBA expression has been linked to environmental stress in plants [52]. This is indicative of variations in the Calvin cycle or glycolysis/gluconeogenesis, which may contribute to an increase in sucrose synthesis, thereby providing more energy for plant metabolism under water-deficit stress conditions [53].

The DNA-directed RNA polymerase beta subunit is an enzyme present in the chloroplasts. Its function is to transcribe DNA into RNA using ribonucleoside triphosphates as substrates. Maturase K is a component of chloroplasts involved in the biological processes of RNA splicing and processing of mRNA and tRNA.

The YABBY 2 protein, exclusively accumulated in the commercial cultivar RB867515, is present in the cell nucleus. It is present in leaf blades, sheaths, and flowers. Through a search of the public database Uniprot (http://www.uniprot.org/uniprot/Q10FZ7), accessed on 25 November 2013, it was observed that this protein is encoded by *YAB2*. Siegfried et al. [54], who studied the YABBY gene family in *Arabidopsis*, reported that its expression is inherently related to the structuring of floral organs. Toriba et al. [55] also studied the YABBY gene family in *Arabidopsis*, starting from the isolation of these genes in rice (*Oryza sativa*). They concluded that in flowers, the expression of these genes is related to the regulation of the differentiation of specific cell types.

In the specific case of sugarcane, flowering is extremely detrimental to commercial plantations because of a phenomenon known as isoporization. This phenomenon is characterized by the dehydration of stalks, resulting in weight loss and a simultaneous decrease in agricultural and commercial productivity. Moreover, isoporization leads to an increase in the fiber content and a reduction in the effectiveness of sugar extraction by mills [56]. Flowering in sugarcane occurs during prolonged dry periods and serves as a plant strategy to ensure the perpetuation of its species when it senses a threat from environmental stress. The absence of this protein in the clone resulting from self-fertilization indicated that it exhibited greater tolerance to water-deficit stress. This protein may represent a candidate for developing functional molecular markers for the selection of genotypes with low flowering rates in breeding programs. Studying proteins related to flowering in sugarcane, particularly in the northeastern region where flowering is intense and detrimental, could help identify such proteins and develop techniques to inhibit their activity. This will contribute to breeding programs and enhance sugarcane productivity.

One of the proteins identified using mass spectrometry was a hypothetical protein. A search in the UniProt database indicated that it is a predicted protein whose function has not been described in the database. Therefore, this is a target for further study and characterization.

According to the UniProt database, the nucleoside diphosphate kinase 1 protein is directly involved in nucleotide metabolism, specifically in the biosynthesis of CTP, GTP, and UTP. This protein was identified by Moisyadi et al. [57] and is expressed by the *NDPKI* gene. In their experiments, these authors increased the activity of this protein in cell extracts exposed to heat, concluding that the response to heat shock involved substantial changes in gene transcription, mRNA translation, protein synthesis, and other metabolic events, leading to the development of thermotolerance. Therefore, the presence of this protein in the commercial cultivar, RB867515, and the clone resulting from self-fertilization, RB061047, explains why, even in the adverse climatic conditions of the sugarcane region in the State of Pernambuco, where temperatures and heat are high, the evaluated genotypes, especially the self-fertilization-derived clone, exhibited accelerated metabolism, better photosynthetic activity, and greater efficiency in water-use under unfavorable cultivation conditions. Additionally, this protein is a highly promising candidate for the development of functional molecular markers applicable to genetic improvement in the development of new genotypes that are tolerant to water stress and high temperatures.

Wang et al. [58] presented the mechanisms of drought response in the leaves of different crops based on proteomic analysis from different studies, making it possible to infer that the proteins ATPA, FBA, YABBY2, and NDPK1 were potential candidates for functional molecular marker development. In addition, these proteins are directly linked to three essential categories related to the water stress response: energy and homeostasis, signaling and defense, and gene regulation and morphology.

Functional molecular markers are directly linked to phenotypic features of interest. Specifically in sugarcane, they are linked to features such as high productivity, sugar content, biomass, and tolerance when cultivated under stress conditions. The identified proteins satisfied these criteria. The ATPA is a potential marker of photosynthetic energy efficiency. Genotypes that maintain high ATPA expression under stress demonstrate a greater capacity to sustain metabolism and produce the necessary energy (ATP) for repair and adaptation [59]. Selecting cultivars with genes or alleles that promote ATPA stability may indicate their superiority in maintaining energy homeostasis, which is crucial for growth and higher sugar yields under water stress.

NDPK1 is a potential functional marker for rapid defense and signaling. This protein is located at the center of signaling cascades and antioxidant defense. Higher expression indicates a more robust alert and defense response [60]. This allows plants to quickly signal stress and mobilize mechanisms to neutralize reactive oxygen species (ROS), which cause cellular damage. In a breeding program, genotypes with higher NDPK1 accumulation/activity are selected for their cellular resilience and ability to prevent oxidative damage, which may lead to a drop in productivity during drought.

FBA has the potential to be a marker for osmotic adjustment and is a control point for the glycolytic and gluconeogenesis pathways [61]. Its differential regulation likely indicates a key alteration in carbon metabolism to divert sugar production towards the synthesis of compatible solutes (such as glucose, sucrose, or proline) that can help the plant retain water through osmotic adjustment. This was observed in clone RB061047, which showed a higher concentration of proline and more efficient water-use under water-deficit conditions than the commercial hybrid.

Finally, the YABBY2 protein is a potential marker for gene regulation associated with morphogenesis. Its exclusive expression in the commercial genotype suggests that this protein might be related to an adaptive response, potentially orchestrating major changes, such as reprogramming of leaf architecture (reduction in area, possibly to avoid transpiration), development of floral organs, or the control of senescence [62]. In the specific case of cultivar RB867515, as discussed above, it has the characteristic of rapid flowering, which is undesirable in the sugarcane industry, given the drastic loss of sugar content in the stalk. However, the high expression of YABBY2 in other genotypes reinforces its use as a potential marker for a sophisticated regulatory response, indicating that the cultivar has the capacity to adjust its development to minimize water loss, which is a highly effective tolerance strategy in the field.

In summary, the accumulation of these proteins seems to be consistent under stress conditions, and they may be considered reliable indicators of water-deficit tolerance, as they represent and modulate essential cellular mechanisms usually associated with drought tolerance. The sugarcane breeding program of RIDESA covers the entire sugarcane-growing region in the state of Pernambuco. This region is characterized by severe environmental variations, including irregular rainfall and long periods of drought. Therefore, with these functional molecular marker candidates, the selection of new sugarcane cultivars for the sugarcane-growing region of the State of Pernambuco (and other regions where water deficit is relevant) can be made based not only on phenotypic tolerance, but also on the presence of specific cellular and genetic mechanisms that confer this tolerance. In hybridization processes, the transmission of the characteristics of interest to new genotypes can be optimized.

Water deficit stress tolerance is a highly complex mechanism involving several proteins. From the perspective of plant breeding, water stress tolerance is controlled by multiple genes, and several challenges need to be overcome. These proteins accumulate at different stages of stress in response to the environmental stimuli. Controlled by quantitative trait loci (QTLs), the expression of respective coding genes may be strongly influenced by subtle environmental variations. Marked differences in protein synthesis may exist among genetically distinct cultivars, making it challenging to identify consistent expression patterns of specific proteins. In sugarcane, this is even more challenging because of the complexity of the genome. Self-fertilization has emerged as a promising strategy; however, further research and applications are necessary for new discoveries, and the potential of self-fertilization needs to be validated in other genotypes subjected to different abiotic stresses.

To date, most experiments have been conducted in greenhouses with strict temperature, irrigation, and environmental control. Additionally, data collection, experimental design, frequency of data collection, and stress exposure follow a rigorous methodology. The question arises as to whether the results obtained under these conditions faithfully reproduce the behavior of genotypes in commercial cultivation settings, where environmental variations and stress exposure levels are much more complex and varied, given that field conditions involve multiple stress factors. Therefore, it is important that new experiments be conducted in the sugarcane regions of Pernambuco within their respective production environments, varied topography, soil types, and different temperature regimes. Comparing the results achieved with those obtained from greenhouse experiments would undoubtedly enhance our understanding of this highly complex mechanism, contributing substantially to the development of genotypes adapted not only to water-stress, but also to various stressors that affect sugarcane productivity.

## 4. Materials and Methods

### 4.1. Plant Material and Growth Conditions

**Figure 2:**
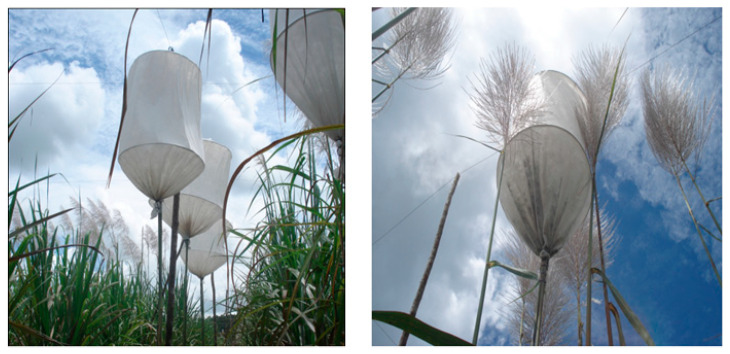
Non-woven fabric bell used for the effective control of self-fertilization in the RB867515 cultivar.

Biochemical and physiological analyses were conducted on two genotypes: the commercial cultivar, RB867515 (tolerant to water deficit stress), and the clone, RB061047, already encoded by PMGCA/UFRPE/RIDESA, resulting from the self-fertilization of the cultivar RB867515 at 8 months of age (Figure 2).

**Figure 3:**
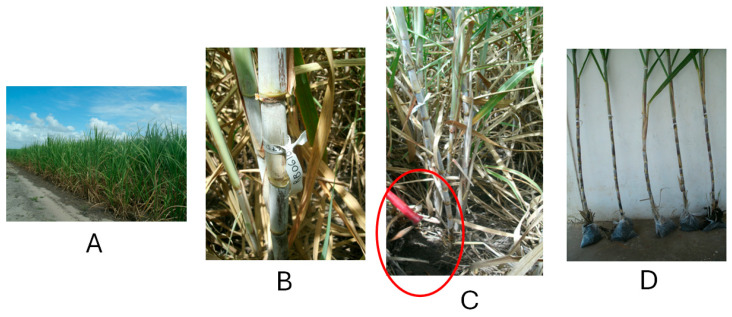
Genotypes cultivated in the agricultural area of the Santa Tereza sugar mill, in the municipality of Goiana, PE. (**A**) identification of the experimental field in the multiplication phase of sugarcane genetic improvement (MP). (**B**) Identification of the genotypes (RB867515 and RB061047) and the repetitions to be selected for analysis. (**C**) Selection of the experimental genotypes, highlighting the collection of soil samples. (**D**) Sample of the genotypes sent for analysis.

The genotypes were evaluated in the multiplication phase (MP) under rainfed conditions in the agricultural area of the Santa Teresa sugarmill, Goiana, PE (Figure 3). Specifically, the study took place at the Japumin mill (07°33′ S, 35°00′ W), which has an altitude of 13 m, a mean temperature of 26 °C, and rainfall of 511.6 mm.

The objective of this phase was to continue selecting the best clones and multiplying the selected genotypes on a larger scale. For the aforementioned analyses, six replicates of each genotype were considered. Soil samples were collected for physical analysis.

### 4.2. Determination of Organic Solutes

For this assessment, approximately 1 g of leaves wrapped in aluminum foil was collected and stored in a −20 °C freezer until the preparation of the extracts. The samples were thawed and macerated in a 0.1 M monobasic potassium phosphate-buffered solution at pH 7.0, and stored in Eppendorf tubes. The extracts were centrifuged at 10,000 rpm for 10 min and transferred to Eppendorf tubes. Organic solutes in the extracts were quantified using absorbance readings on a spectrophotometer.

Total free carbohydrates were determined using the phenol–sulfuric acid method [63]; soluble protein content was determined using the Coomassie Brilliant Blue dye-binding method [64]; amino acid content was determined using the ninhydrin method [65]. The concentration of free proline was determined using the ninhydrin and phosphoric acid methodology proposed by Bates et al. [65], with absorbance readings at 490, 595, 570, and 520 nm for each applied methodology. Concentrations were expressed in μmol.g^−1^ MF. Samples were collected for biochemical analyses at 12 PM.

### 4.3. Determination of Photosynthetic Pigments

The chlorophyll readings were obtained using a portable SPAD-502 device (Minolta, Japan). The obtained value was the average of 10 readings taken from leaves spread across the plant between 11 AM and 12 PM for each genotype. Physiological measurements were taken at this time because of stomatal opening. During this period, the evaporative demand was higher, as determined by the daily photosynthesis curve at 7, 9, 11, and 13 h.

Chlorophyll and carotenoid contents were determined with 0.1 g of fresh leaf material from each replicate. These samples were then placed in threaded test tubes covered with aluminum foil to prevent light passage, and 10 mL of 95% ethyl alcohol was added. After a 48 h period, the contents of chlorophyll a, b, total chlorophyll, and carotenoids were quantified following the methodology of Lichtenthaler and Buschmann [66]. The quantification was performed using a Biospectro spectrophotometer (model SP-220), with readings taken at wavelengths of 664 nm, 649 nm, and 470 nm. The data were calculated using the formulae below; results are expressed in milligrams of chlorophyll per gram of fresh tissue (mg·g^−1^):

Chla (μg/mL) = 13.36 × A 663.1 − 5.19 × A 648.6(1)

Chlb (μg/mL) = 27.43 × A 648.6 − 8.12 × A 664.1(2)

Chltotal (μg/mL) = Clor a + Clor b(3)

Carotenoids (μg/mL) = [1000 × A_470_ − 2.13 (Cl a) − 97.64 (Cl b)/209] (4)

### 4.4. Gas Exchange: Liquid Photosynthesis, Transpiration, Stomatal Conductance, and Water-Use Efficiency

Gas exchange measurements were conducted between 11:00 AM and 1:00 PM following the differentiation of the treatments. Photosynthesis (A), transpiration (E), and stomatal conductance (gs) were assessed using an infrared gas analyzer (ADC, model LcPro). From these results, it was possible to calculate the water use efficiency using Equation (5). Leaves +1, which were fully expanded and located in the upper-middle third of the plants, were used for the measurements.

WUE = A/E(5)

Physiological measurements were taken at this time because of the opening of the stomata, during which evaporative demand was higher, as determined by the daily photosynthesis curve at 07:00, 09:00, 11:00, and 13:00.

### 4.5. Quantum Efficiency of Photosystem II

The quantum efficiency of Photosystem II was determined using a portable light fluorometer (OptiSciences, Hudson, NY, USA). This determination relied on the relationship between initial fluorescence (IF) and maximum fluorescence (MF) after the leaves had adapted to darkness for 30 min. All biochemical and physiological analyses were conducted at the Plant Physiology Laboratory of the Department of Biology, Federal Rural University of Pernambuco (UFRPE). The physiological and biochemical data were subject to analysis of variance, and the means were compared using Tukey’s test at a 5% probability level.

The analysis of variance was performed according to statistical completely randomized linear additive model:

(6) Yij = µ + Gi + Ɛij

where Yij: is the observation of the ith genotype in the jth replicate; µ is the overall mean; Gi is the effect of ith genotype; and Ɛij is the random error.

The effects of the mean and genotypes were considered fixed, whereas the experimental error was considered random. Therefore, the statistical model used was classified as a fixed effects model. All statistical analyses were performed using R software version 3.6.3 [67].

### 4.6. Proteomic Analysis

For proteomic analysis, +1 leaves were collected following the method of van Dillewijn [68], which corresponded to the first leaf from top to bottom with a visible sheath. The collected leaf samples were washed with distilled water, cut into smaller fractions with scissors previously cleaned with 70% alcohol, and placed in 50 mL conical tubes (Falcon). Subsequently, they were immediately immersed in liquid nitrogen and transported to the Plant Genomics and Proteomics Laboratory at Federal University of Pernambuco (UFPE), where they were stored at −80 °C.

Total proteins from the sugarcane genotypes were extracted using the method described by Hurkman and Tanaka [69], which utilized phenol as the primary extraction reagent. Plant material samples were ground in a porcelain mortar with a pestle in liquid nitrogen until a fine powder was obtained, which was then stored in 50 mL Falcon tubes. Approximately 1 g of the ground plant material was used for extraction, conducted by adding 500 μL of extraction buffer composed of Tris HCl (pH 8.5; 0.1 M), EDTA (10 mM), sucrose (0.9 M), 2-β mercaptoethanol (0.4%), and 500 μL of phenol. The samples were vigorously shaken for 1 min without heating, and then kept under constant agitation for an additional 30 min at 4 °C. After this step, centrifugation was carried out at 5000× *g* for 10 min at 4 °C, and the supernatant was transferred to a new tube.

Re-extraction was conducted where the previous process was repeated, but without the 30-minshaking step, and centrifugation was changed to 12,000× *g* for 20 min. Proteins were precipitated by adding five volumes of ice-cold ammonium acetate in methanol (0.1 M) and incubated at −20 °C for 24 h. The samples were centrifuged at 15,000× *g* for 20 min at 4 °C, the supernatant was discarded, and two washes of the pellet were conducted with 1 mL of ammonium acetate in methanol (0.1 M) and 80% acetone, and one wash with 70% ethanol, interspersed with incubation steps for 30 min at −20 °C and centrifugation (15,000× *g*, 10 min at 4 °C). After the washes, the pellet was dried at room temperature, and the proteins were solubilized in a urea and thiourea solution (8 M:2 M), and incubated at −20 °C for subsequent 2D analysis.

### 4.7. Protein Quantification

Total soluble proteins were quantified using the Bradford method [63], which used Coomassie Brilliant Blue G-250 as the staining reagent and bovine serum albumin (BSA) to establish a standard curve. Deionized water and the staining reagent were added to 20 μL of the protein samples to reach a final volume of 2 mL, which was then analyzed in a visible light spectrophotometer (BioSpectro SP-220) at 595 nm. Protein integrity was visualized using 12.5% sodium dodecyl sulfate-polyacrylamide gel electrophoresis.

### 4.8. Two-Dimensional Electrophoresis

#### 4.8.1. Isoelectric Focusing (IF)—1st Dimension

IF was conducted using 11 cm IPG strips at a pH range of 3–10 for each of the three biological replicates. A total of 60 μg of total leaf protein samples was applied to dehydrated acrylamide strips (11 cm IPG, nonlinear pH 3–10 gradient; GE Life Sciences) dissolved in a solution of 7 M urea, 2 M thiourea, 2% (*w*/*v*) CHAPS, 19.4 mM DTT, 0.5% (*v*/*v*) ampholytes (IPG buffer, nonlinear pH 3–10; GE Life Sciences, Marlborough, MA, USA), and 0.005% (*w*/*v*) bromophenol blue. The strips were then rehydrated using an isoelectric focusing device (IPGphor; GE Life Sciences, Marlborough, MA, USA) at room temperature for 16 h. The strips were focused on an immobilized pH gradient in three steps under the following conditions: 300 V, 30 V/h; 3500 V, 2900 V/h; and 3500 V, 6170 V/h. Throughout the focusing process, constant current (2 mA) and power (5 W) were maintained. After focusing, the strips were stored at −20 °C until the second dimension was conducted.

#### 4.8.2. Electrophoresis—2nd Dimension

Following isoelectric focusing, the IPG strips were equilibrated with a solution containing 50 mM Tris-HCl (pH 8.8), 6 M urea, 30% (*v*/*v*) glycerol, and 2% (*w*/*v*) SDS in two steps. A 1% (*w*/*v*) DTT was added and shaken for 20 min, followed by the addition of 2.5% (*w*/*v*) IAA and shaken for another 20 min. The second dimension of electrophoresis was carried out using the Multiphor II Electrophoresis Unit (GE Life Sciences) on a 12.5% polyacrylamide gel, ExcelGel homogeneous (GE Life Sciences) of 24 cm at 15 °C. The first stage of electrophoresis was conducted at 120 V, 20 mA, and 30 W under constant conditions for approximately 40 min. This stage concluded when the sample diffused from the IPG strip into the gel. The second stage of the run was conducted at 600 V, 50 mA, and 30 W.

### 4.9. Gel Staining

After electrophoresis, the gels were placed in a fixing solution (40% ethanol and 10% acetic acid) for 30 min, followed by staining (Coomassie PhastGel Blue R dye, GE Life Sciences) at 0.02% in methanol, 49% acetic acid, and deionized water at a ratio of 3:1:6 for 1.5 h. The gel was destained after four consecutive washes with destaining solution (20% ethanol and 5% acetic acid) for 15 min and 45 min, and twice for 2 h. After washing, the gels were stored in preservation solution (5% acetic acid).

### 4.10. Image Acquisition

After destaining, the gels were scanned using ImageScanner III (GE Life Sciences) with LabScan 6.0 program (GE Life Sciences). The following parameters were used for scanning: transparency mode, 150 dpi resolution, and a red filter, which is ideal for Coomassie blue-stained gels. Prior to image capture, the scanner was calibrated using a Kodak 3 calibration strip, which provided defined optical or diffuse density values, following the manufacturer’s recommended procedures.

### 4.11. Image Analysis for Spot Detection

The gel images were analyzed using Image Master 2D Platinum v7.05 software (GE Life Sciences). The images were processed using the program and adjustments regarding the size and reduction in contrast interference were made. Projects were created in which the gels were grouped into folders according to the program’s recommended hierarchies. Comparisons (matches) were then conducted within these folders.

To enable statistical inferences, the pH gradient values from DryStrip and the molecular mass values of the bands generated by the marker used were registered in the program. Three biological replicates were used for drought conditions and analyses. Initially, the correlation coefficients among biological replicates were observed to assess gel reproducibility. Analyses were conducted only if the biological replicates exhibited a high correlation coefficient. Based on normalization and statistical analyses performed using the Image Master 2D Platinum v7.05 software (GE Life Sciences), it was possible to identify differentially expressed proteins, as well as exclusive spots between treatments and varieties, potentially associated with responses to water stress. These spots were selected for subsequent digestion and identification using mass spectrometry.

The results were presented in the form of various gel analysis reports and comparisons (matches) within the program. These reports provided important information about the spots, including their location in the gel, isoelectric point (pI), molecular weight (MW), and percentage volume (%), as well as details on comparisons (matches) and statistical inferences, such as the % volume ratio (Ratio), which represents the ratio between two corresponding spots in a match, and the ANOVA value. Spots with Ratio ≥ 1.5 and ANOVA ≤ 0.05 were selected, ensuring statistical reliability for this analysis. These criteria were applied to the common spots in the comparisons. For exclusive spots, only ANOVA ≤ 0.05 was considered.

### 4.12. Trypsin Digestion

The methodology described by Pacheco et al. [45] was used for spot digestion. The spots were manually excised from the gels, properly identified by gel and match of origin, and stored at −20 °C in sterile deionized water. Initially, the gel fragments were decolored by incubation in 50% methanol/2.5% acetic acid for 3 h at room temperature. After dehydration with 100% acetonitrile, the proteins were reduced with 10 mM DTT (dithiothreitol) solution, followed by alkylation with a 50 mM IAA solution. The gel was washed with 100 mM ammonium bicarbonate and dehydrated with 100% acetonitrile, and this process was repeated once. After these steps, a solution containing 20 μg of trypsin was added for protein digestion, and it was incubated on ice for 30 min. The excess trypsin was removed, and a 50 mM ammonium bicarbonate solution was added, covering the gel and incubating it for 16 h at 37 °C. The following day, without removing the previous solution, extraction solution 1 (5% formic acid) was added, and the mixture was incubated for 10 min at room temperature. After centrifugation, the supernatant was transferred to another tube. The same procedure was performed for extraction solution 2 (5% formic acid in 50% acetonitrile) and repeated twice. Finally, the samples were completely evaporated using speed-vac centrifugal evaporators (Eppendorf) and stored at −20 °C until analysis using mass spectrometry.

### 4.13. Spot Protein Analysis

Proteins were analyzed using an AutoFlex III TOF/TOF mass spectrometer (Bruker Daltonics, Bremen, Germany) based on matrix-assisted laser desorption/ionization. For this purpose, peptides, after concentration and drying, were eluted in a 1% α-cyano-4-hydroxycinnamic acid matrix solution. The MS spectra were obtained following the parameters of the reflected method, RP_Proteomics, calibrated in the equipment, excluding ions with *m*/*z* less than 700 Da. After obtaining the MS spectra, peaks with a higher intensity (greater than 3000) were fragmented using the LIFT method in the presence of argon. The obtained spectra were analyzed and sequenced using FlexAnalysis Software, considering the mass-to-charge ratio (*m*/*z*) mass table.

### 4.14. Protein Identification

MS spectra were obtained on an Autoflex III MALDI-ToF/ToF spectrometer (Bruker Daltonics, Billerica, MA, USA) available at the Center for Strategic Technologies of the Northeast, CETENE, Recife, PE, using α-cyano-4-hydroxycinnamic acid matrix (Sigma/C8982) and according to the standardized analysis protocols of peptide mass fingerprinting (PMF; positive reflected method RP_Proteomics_HPC). Ions were accelerated at 19 KV, with a Nd:YAG smart beam 355 nm laser at 100 Hz. For MS, the spectra were identified by analyzing the peaklist.xml files in Mascot software (Matrix Inc., Jersey City, NJ, USA) using the PMF against the plant database (Viridiplantae) available in the online version of Mascot (http://www.matrixscience.com) accessed on 25 November 2013, using the following parameters: database, Swissprot; taxonomy, Viridiplantae; fixed modification, carbamidomethyl (C); variable modification, oxidation (M); and tolerance, 200 ppm. Identification with a score greater than the cutoff value was considered significant. The score is equivalent to −10. log(P), where P is the probability that the similarity identified is a chance. Scores above the cutoff value were considered statistically significant (*p* < 0.05). The significantly identified accessions were searched in the UniProt database (http://www.uniprot.org/) accessed on 25 November 2013 for details of the annotated protein descriptors.

## 5. Conclusions

Clone RB061047 did not exhibit signs of inbreeding depression; instead, it demonstrated improved physiological performance under water-deficient conditions. This is possibly related to higher FBA accumulation.

The identification of ATPA, YABB2, FBA and NDPK 1 seems to be associated with the mechanism of tolerance to water deficit stress. These proteins are promising candidates for the development of functional molecular markers for identifying and developing water deficit-tolerant genotypes.

## Figures and Tables

**Table 1:** Physical analysis of the soil samples from the experiment conducted during the multiplication phase of sugarcane breeding, carried out at the agricultural area of the Santa Tereza sugarmill (Goiana-PE), in the 2012/2013 crop season.

SAMPLE	D (cm)	SD (g/cm^3^)	PD (g/cm^3^)	TP (%)	NC (%)	FD (%)	TS (%)	CS (%)	FS (%)	S (%)	C (%)	FC (mg/mg)	PWP (mg/mg)	AW (mg/mg)
1	00–20	1.36	2.63	48.43	3.04	74.31	81.04	59.00	22.04	7.13	11.83	0.065	0.023	0.042
1	20–40	1.33	2.63	49.36	3.04	80.80	81.84	63.40	18.44	2.33	15.83	0.070	0.040	0.030
2	00–20	1.36	2.67	48.96	3.04	78.14	81.28	55.16	26.12	4.82	13.90	0.063	0.028	0.034
2	00–40	1.36	2.56	47.15	3.04	78.14	80.48	50.86	29.62	5.62	13.90	0.055	0.030	0.025

D = depth; SD = soil density; PD = particle density; TP = total porosity; NC = natural Clay; FD = flocculation degree; TS = total sand; CS = coarse sand = FS = fine sand; S = silt; C = clay; FC = field capacity; PWP = Permanent wilting point; AW = available water.

**Table 2:** Biochemical and physiological analyses of the RB867515 cultivar and the RB061047 clone, evaluated under dryland conditions during the multiplication phase (MP) at the agricultural area of Santa Teresa (Goiana—PE), in the 2012/2013 crop season.

Organic Solutes						
Genotypes	Amino Acids (µmol·gMF^−1^)	Carbohydrates (µmol·gMF^−1^)		Proline (µmol·gMF^−1^)	Protein (µγ·gMF^−1^)	
RB867515	9.47 a	128.92 a		0.098 b	332.49 a	
Standard deviation	0.80	26.98		0.01	65.89	
RB061047	7.75 ab	94.77 b		0.130 a	201.69 ab	
Standard deviation	3.06	20.38		0.02	208.12	
F values	0.15 ^ns^	28.60 **		7.50 *	0.39 ^ns^	
DF Genotypes	1	1		1	1	
DF Residual	10	10		10	10	
DF Total	11	11		11	11	
Trend	ns	-		+	ns	
Pigments						
Genotypes	Chlorophyll a (mg·g^−1^)	Chlorophyll b (mg·g^−1^)	Total Chlorophyll (mg·g^−1^)	Carotenoids (mg·g^−1^)	SPAD wp	SPAD Leaves+1
RB061047	1.91 a	1.79 a	3.72 a	0.70 a	32.74 a	32.90 a
Standard deviation	0.15	0.31	0.44	0.03	3.75	3.60
RB867515	1.97 a	1.75 a	3.73 a	0.74 a	36.18 a	33.78 a
Standard deviation	0.21	0.35	0.57	0.05	2.59	3.43
F values	0.26 ^ns^	0.06 ^ns^	0.0003 ^ns^	1.78 ^ns^	2.85 ^ns^	0.16 ^ns^
DF Genotypes	1	1	1	1	1	1
DF Residual	10	10	10	10	10	10
DF Total	11	11	11	11	11	11
Trend	ns	ns	ns	ns	ns	ns
Photosynthesis						
Genotypes	Leaf+1 Transpiration (E, µmol H_2_O m^−2^·s^−1^)	Stomatal Condutance (*g_s_*, mol·m^−2^·s^−1^)		Liquid Photosynthesis (A, µmol·m^−2^·s^−1^)	Water Use Efficience (WUE, µmol CO_2_ mmol H_2_O)	
RB061047	5.40 a	0.36 a		24.01 a	4.65 a	
Standard deviation	0.92	0.11		5.84	1.42	
RB867515	5.80 a	0.22 a		16.30 b	2.84 b	
Standard deviation	1.24	0.11		2.29	0.44	
F values	0.71 ^ns^	4.35 ^ns^		7.74 *	7.61 *	
DF Genotypes	1	1		1	1	
DF Residual	10	10		10	10	
DF Total	11	11		11	11	
Trend	ns	ns		+	+	
qP Photosystem II						
Genotypes	F_0_		F_m_		F_v_/F_m_	
RB061047	48.00 b		221.22 b		0.78 a	
Standard deviation	5.12		17.53		0.02	
RB867515	57.00 a		253.00 a		0.77 a	
Standard deviation	4.72		14.37		0.01	
F values	16.64 **		716.26 **		0.01 ^ns^	
DF Genotypes	1		1		1	
DF Residual	10		10		10	
DF Total	11		11		11	
Trend	-		-		ns	

Trend column refers to significant parameter increase or decrease in the self-fertilized clone compared to the hybrid cultivar. Analyte content or parameters and respective units are: Amino Acids (µmol·gMF^−1^); Carbohydrates (µmol·gMF^−1^); Proline (µmol·gMF^−1^); Protein (µγ·gMF^−1^); Chlorophyll a (mg·g^−1^); Chlorophyll b (mg·g^−1^); Total Chlorophyll (mg·g^−1^); Carotenoids (mg·g^−1^); SPAD Whole Plant; SPAD Leaf+1; Leaf+1 Transpiration (µmol H_2_O m^−2^·s^−1^); Stomatal Conductance (*g_s_*, mol·m^−2^·s^−1^); Liquid Photosynthesis (A, µmol.m^−2^·s^−1^); and Water Use Efficiency (WUE, µmol CO_2_. mmol H_2_O); Initial Fluorescence (F_0_); Maximum Fluorescence (F_m_, RU) and Quantum Efficiency of Photosystem II (qP, F_v_/F_m_); DF, Degrees of Freedom; Wp, Whole Plant. ** or * significant at the 1% or 5% probability level by the F-test; ns, not significant. Means followed by the same letters in a column do not differ statistically according to Tukey’s test at 5% probability level.

**Table 3:** Identification of differentially expressed proteins in different sugarcane genotypes under water-deficit stress, showing expression in the hybrid cultivar, RB867515 (H) or in the self-fertilization-derived clone, RB061047 (S).

Spot/Match ID	Ratio	*p*-Value	Protein ID	Accession	Score	Orthologous Species
Common						
328/24	1.65 (S and H)	0.035	Fructose-bisphosphate aldolase	P08440	74	*Zea mays*
244/51	5.61 (S and H)	0.032	ATP synthase alpha subunit	P05022	118	*Zea mays*
445/81	6.45 (S and H)	0.042	Nucleoside-diphosphate kinase 1	P93554	ns	*Saccharum officinarum*
Exclusive						
216/130	Excl. (H)	0.014	YABBY 2 protein	Q10FZ7	63	*Oryza sativa subsp. japonica*
330/113	Excl. (H)	0.051	DNA-dependent RNA polymerase beta subunit	H9LGX	58	*Gossypium hirsutum*
291/114	Excl. (H)	0.035	Hypothetical protein	-	75	*Volvox carteri f. nagariensis*
163/131	Excl. (H)	0.035	Maturase K	Q6ENX8	ns	*Saccharum officinarum*

Spot/Match ID: Identification of the spot on the two-dimensional polyacrylamide gel electrophoresis (2D-PAGE) image. Ratio: Percentage volume variation in the spot between genotypes. ANOVA: *p*-value. ns: Not significant. Protein ID: Identification as listed in the MASCOT Viridiplantae Swiss-Prot database. UniProt Accession: UniProt accession number. Score: MASCOT significant score. Orthologous species: Species with the highest sequence similarity.

## Data Availability

The original contributions presented in this study are included in the article.
